# Separation of bimodal fMRI responses in mouse somatosensory areas into V1 and non-V1 contributions

**DOI:** 10.1038/s41598-024-56305-w

**Published:** 2024-03-15

**Authors:** Thi Ngoc Anh Dinh, Hyun Seok Moon, Seong-Gi Kim

**Affiliations:** 1 Center for Neuroscience Imaging Research (CNIR), Institute for Basic Science (IBS), Suwon, 16419 South Korea; 2 Department of Biomedical Engineering, Sungkyunkwan University, Suwon, 16419 South Korea; 3 Department of Intelligent Precision Healthcare Convergence, Sungkyunkwan University, Suwon, 16419 South Korea

**Keywords:** Neuroscience, Sensory processing, Somatosensory system, Visual system

## Abstract

Multisensory integration is necessary for the animal to survive in the real world. While conventional methods have been extensively used to investigate the multisensory integration process in various brain areas, its long-range interactions remain less explored. In this study, our goal was to investigate interactions between visual and somatosensory networks on a whole-brain scale using 15.2-T BOLD fMRI. We compared unimodal to bimodal BOLD fMRI responses and dissected potential cross-modal pathways with silencing of primary visual cortex (V1) by optogenetic stimulation of local GABAergic neurons. Our data showed that the influence of visual stimulus on whisker activity is higher than the influence of whisker stimulus on visual activity. Optogenetic silencing of V1 revealed that visual information is conveyed to whisker processing via both V1 and non-V1 pathways. The first-order ventral posteromedial thalamic nucleus (VPM) was functionally affected by non-V1 sources, while the higher-order posterior medial thalamic nucleus (POm) was predominantly modulated by V1 but not non-V1 inputs. The primary somatosensory barrel field (S1BF) was influenced by both V1 and non-V1 inputs. These observations provide valuable insights for into the integration of whisker and visual sensory information.

## Introduction

The integration of diverse sensory inputs is crucial for animals to enhance survival opportunities by enriching behavioral outcomes in the real world. In particular, mice acquire information on their surrounding environment relying on whisker sensation in combination with other sensory modalities, such as vision. The convergence of information across the senses takes place at various brain regions along the sensory tracts, which are established by multiple conventional electrophysiological recordings and optical imaging studies^1–11^. Previous studies have reported that not only the posterior parietal association (PTLp)^8^ and the superior colliculus (SC)^5,11,12^, but also the primary somatosensory barrel field (S1BF)^7,9^ and ventral posteromedial thalamic nucleus (VPM)^1,3^ exhibit enhanced responses to bimodal stimulation compared to unimodal whisker stimulation.

Tracing studies have shown reciprocal but asymmetric anatomical connections between visual and somatosensory cortices, where the primary visual cortex (V1) has a stronger anterograde connection to the S1BF^7^, suggesting the involvement of V1 may exert influence on the whisker-sensory information processing such as provide guidance information for tactile sensing or navigation by whisker. In addition, electrophysiological studies reported that stimulation of V1 recruits neural activity in the VPM, the first-order thalamus in whisker-sensory pathways^1^. The transmission of visual inputs to whisker-related areas is likely relayed by V1. Other possible pathways could be the multimodal subcortical nuclei, such as the SC and the lateral posterior thalamic nucleus (LP). Anatomical tracing studies have revealed extensive somatosensory cortical projections from the SC^5,13,14^, which relay more than eighty percent of visual inputs in colliculo-cortical visual pathway^15,16^. The LP is a higher order visual thalamic structure, which has been established as a hub-like center extensively implicated in a broad range of function across various sensory modalities^17^. Overall, the convergence of visual inputs in whisker-related areas involves two main nodes: V1 and subcortical areas.

To investigate long-range interactions between visual and somatosensory processing, we performed blood oxygenation level-dependent (BOLD) fMRI of mice at 15.2 T. Initially, we compared BOLD fMRI responses to unimodal whisker and visual stimulation with bimodal whisker-visual stimulation. Bimodal stimulation enhanced BOLD fMRI responses in whisker cortices and subcortical regions compared to whisker-only stimulation, and multisensory visual-related regions compared to visual-only stimulation. Additionally, BOLD fMRI responses to whisker stimulus were observed in different thalamic regions located in subcortical areas including anterior pretectal nucleus (APN), midbrain reticular nucleus (MRN), substantia nigra (SNr), zona incerta (ZI), parafascicular nucleus (PF), and mediodorsal thalamic nucleus (MD). To investigate the contribution of V1 vs. subcortical related pathways to whisker processing, we performed fMRI of sensory stimulation with silencing of V1 by optogenetic activation of inhibitory neurons^18,19^. We found that VPM predominantly receives the signals from subcortical visual areas, while the posterior medial complex thalamic nucleus (POm) is mostly modulated by V1 but not subcortical signals. Somatosensory cortices such as the S1BF and the secondary somatosensory area (S2) are modulated by both.

## Results

### Unimodal versus bimodal sensory stimulation: whisker, visual versus whisker-visual stimulation

**Figure 1:**
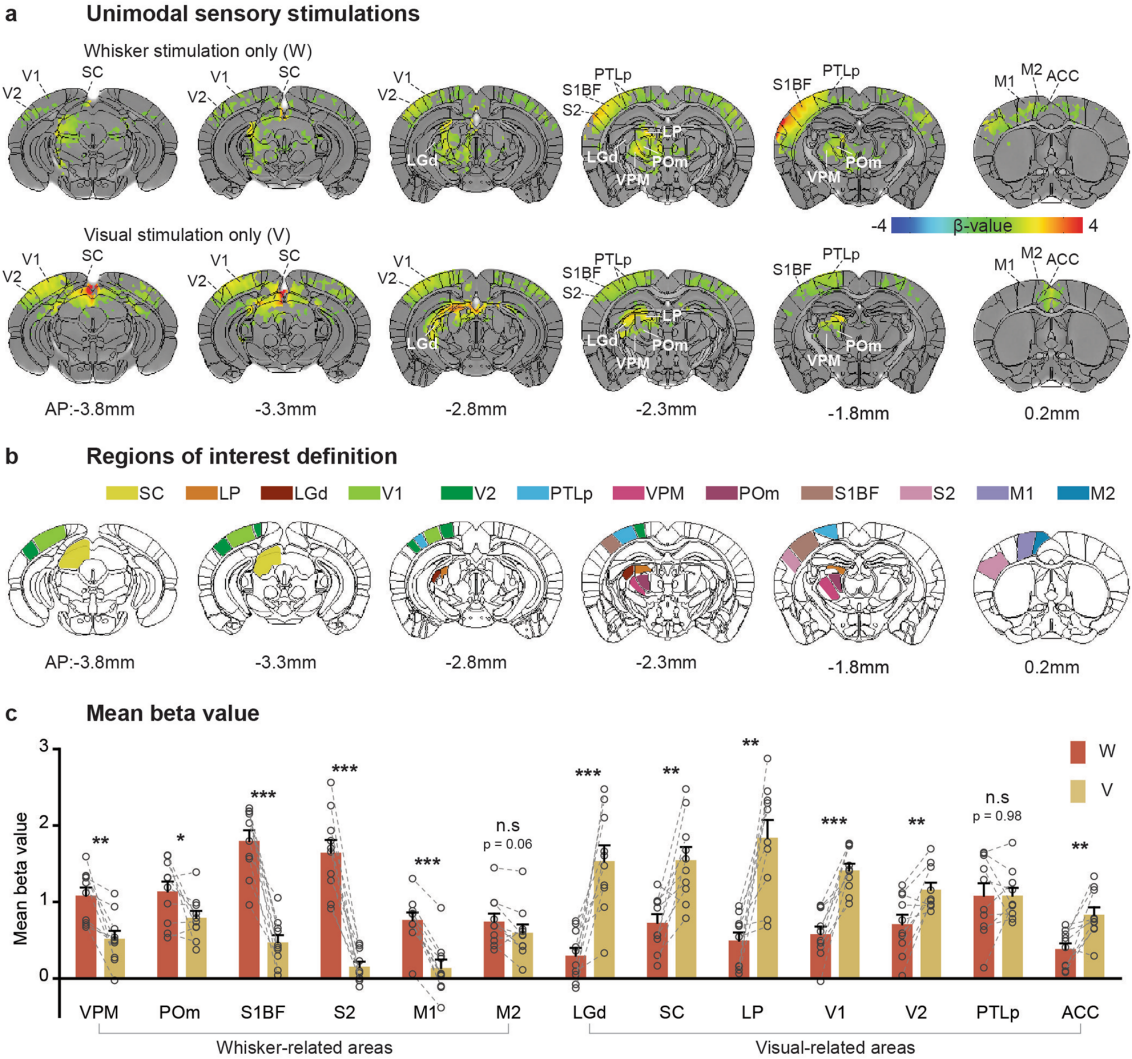
Brain-wide BOLD fMRI responses to right whisker pad and right eye stimulation. Ten mice were used for both unimodal stimulations. (**a**) Group activation maps of the whisker and visual stimulations overlaid on Allen Mouse Brain Atlas (p < 0.01, FDR corrected). Whisker and visual-related areas are labeled for better visualization, and the coronal slice positions are marked relative to Bregma. (**b**) Regions of interest for further quantification. (**c**) Averaged beta values of activation within each ROI, representing the magnitude of the functional response. Each point indicates individual animal’s data. Error bars, SEM; n.s., not significant; *p < 0.05; **p < 0.01; ***p < 0.001 (paired t-test).

Whole-brain BOLD fMRI of lightly anesthetized mice was obtained at an ultrahigh field of 15.2 T for enhancing functional sensitivity^20^. As a reference for bimodal stimulation, we initially acquired fMRI data responding to single sensory whisker (defined as to W) and visual stimulation (defined as to V) in ketamine/xylazine anesthetized mice. Subsequently, we computed group-averaged BOLD fMRI maps using the general linear model (GLM) analysis, and averaged beta values within each region of interest (ROI) for individual mice (Fig. 1).

BOLD fMRI maps of whisker and visual stimulation (Fig. 1a) agree well with observations from previous mouse fMRI studies^15,21,22^. The right whisker pad stimulation evoked robust and localized responses in whisker-related areas, including somatosensory thalamus VPM/POm, S1BF, S2 and motor areas (primary and secondary motor cortex, M1 and M2) (Figs. 1a,c)^22–24^. Widespread BOLD responses were also observed in visual-related areas, with particularly significant activity in the SC, LP, higher-order visual cortex (V2), and PTLp (Figs. 1a,c) as expected due to their multimodal integration role. Specifically, the SC is functionally involved in multisensory integration of sensorimotor and visual systems^4,11,12,22^; the LP is known as part of extrageniculate visual pathway that shares reciprocal connection with multiple sensory areas^10,17,25,26^, and the PTLp is well-established as a mouse visuotactile area^8^. While the activation of other visual areas may be attributed to responses originating from the SC and LP.

Monocular stimulation of the right eye elicited robust BOLD responses across contralateral visual pathway-related areas, the dorsal lateral geniculate thalamic nucleus (LGd), SC, and LP, and in visual cortices consisting of V1 and V2 (Figs. 1a,c)^15,21^. Additionally, pronounced responses in somatosensory networks were observed including the sensory thalamus (VPM and POm) as well as the sensory cortices (S1BF and M2 as illustrated in Fig. 1a,c).

Overall, unimodal sensory stimulation activates the main pathway related to its specific function (e.g., somatosensory-related areas responding to whisker stimulation, visual-related areas responding to visual stimulation), and extends broadly to other functional sites. In this context, the PTLp responds to both stimulations equally (Fig. 1c).

**Figure 2:**
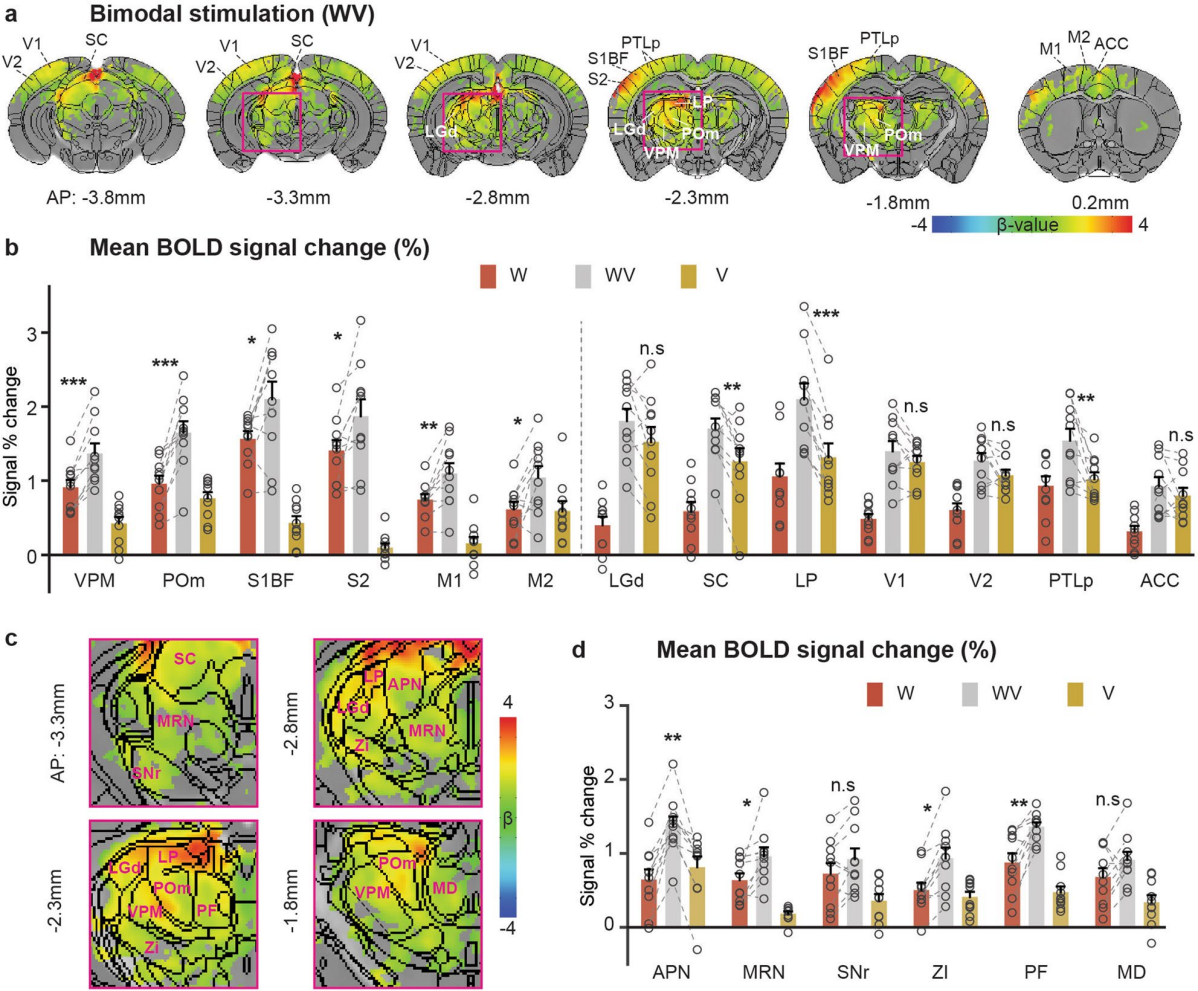
Brain-wide BOLD fMRI responses to bimodal stimulation vs. unimodal stimulation. (**a**) Group activation maps of bimodal stimulation (combination of the right eye and right whisker pad stimulation) overlaid on Allen Mouse Brain Atlas (n = 10; p < 0.01, FDR corrected). Whisker and visual-related areas are labeled for better visualization, and the coronal slice positions are marked relative to Bregma. (**b**) Mean BOLD signal change (n = 10 mice) in the contralateral sensory ROIs responding to whisker-only stimulation (red bar), bimodal stimulation (light gray bar), and visual-only stimulation (yellow bar). During bimodal stimulation, BOLD responses in all somatosensory ROIs were augmented, whereas only multimodal ROIs related in visual pathway were increased (SC, LP, and PTLp). Each circle indicates individual animal’s data, and the same animal data were connected. (**c**) Zoom of the pink ROIs shown in (**a**). (**d**) Mean BOLD signal changes (n = 10) of subcortical ROIs in response to W, WV, and V stimulation. Error bars, SEM; *n.s.* not significant; *p < 0.05; **p < 0.01; ***p < 0.001 (one-way repeated ANOVA).

To investigate cross-modal integration, fMRI was also obtained during the simultaneous application of whisker pad and visual stimuli (WV) (Fig. 2a). BOLD responses of the bimodal stimulus (WV) were compared to mean BOLD responses of the most effective single-modal stimulus (W or V)^27–30^ (Fig. 2b). In comparison to the whisker stimulus (W), bimodal stimulation yielded (WV) significantly higher BOLD responses across most whisker somatosensory related areas (one-way repeated ANOVA, p < 0.05) (Fig. 2b). Similarly, the bimodal stimulation induced higher responses in higher-order visual regions, including the SC, LP, and higher association cortex PTLp compared to visual stimulation (V) (Fig. 2b).

In addition to expected BOLD responses in specific regions, bimodal stimulation induced widespread activities in subcortical regions such as APN, MRN, SNr, ZI, PF and MD (Fig. 2c). The APN, ZI and PF were activated not only by whisker stimulus but also by visual stimulus, while the MRN, SNr and MD mostly responded to whisker stimulation (Supplementary Fig. 1 for time courses). The SNr and MD receive somatosensory inputs via the striatum or prefrontal cortex from the S1BF for further sensory processing^31,32^. We observed significant increases in APN, MRN, ZI and PF activities under the bimodal stimulation compared to whisker-only stimulation (Fig. 2d), indicating the involvement of these subcortical regions in multimodal integration.

**Figure 3:**
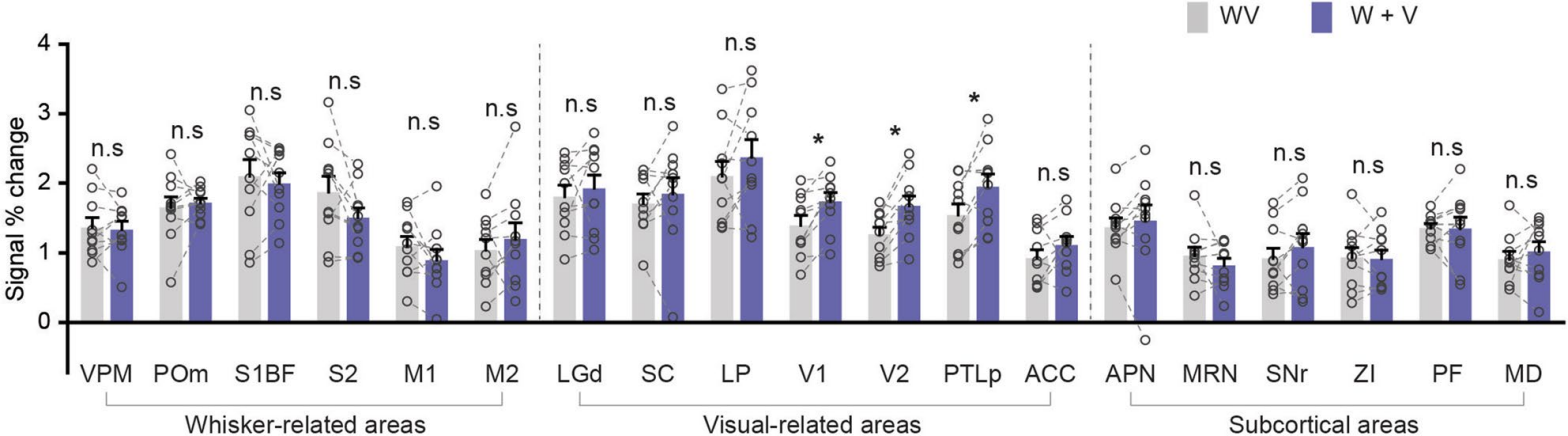
Comparison of mean BOLD changes (n = 10 mice) in the left whisker somatosensory, visual-related, and subcortical ROIs between the bimodal stimulus [WV] (light gray bar) and the sum of unimodal stimulus responses [W] + [V] (blue bar). Error bars, SEM; n.s., not significant; *p < 0.05 (paired t-test).

The enhancement of bimodal stimulation could be different types of effects, such as sub-additive (bimodal < sum of unimodals), additive (bimodal = sum of unimodals), super-additive (bimodal > sum of unimodals), or inhibitory (bimodal < most effective unimodal)^30^. Although fMRI measures hemodynamic responses derived from a population of heterogenerous excitatory and inhibitory neurons^33,34^, we analyzed our bimodal BOLD fMRI data similar to neural data analyses^30^. Mean BOLD responses of whisker-related areas under bimodal stimulation [WV] were compared to the summation of unimodal stimulation responses [W] + [V] (Fig. 3 and Supplementary Fig. 2 for [WV]—([W] + [V])). The results suggest that the enhancement of activation in whisker-related regions by bimodal stimulation can be described by an additive model, where the response to bimodal stimulation [WV] equals the sum of unimodal stimulation responses [W] + [V]. However, in V1, V2 and PTLp bimodal responses appear to be sub-additive, indicating response depression^30^.

In general, bimodal stimulation had distinct effects on different sensory activities, with visual inputs appearing to enhance whisker areal activity by bimodal whisker-visual integration. Meanwhile, whisker inputs only affected multisensory-related area activity in visual-related areas. Thus, whisker-visual integration in the somatosensory regions was further investigated.

### Visual-whisker potential pathways investigated by fMRI with V1 silencing

**Figure 4:**
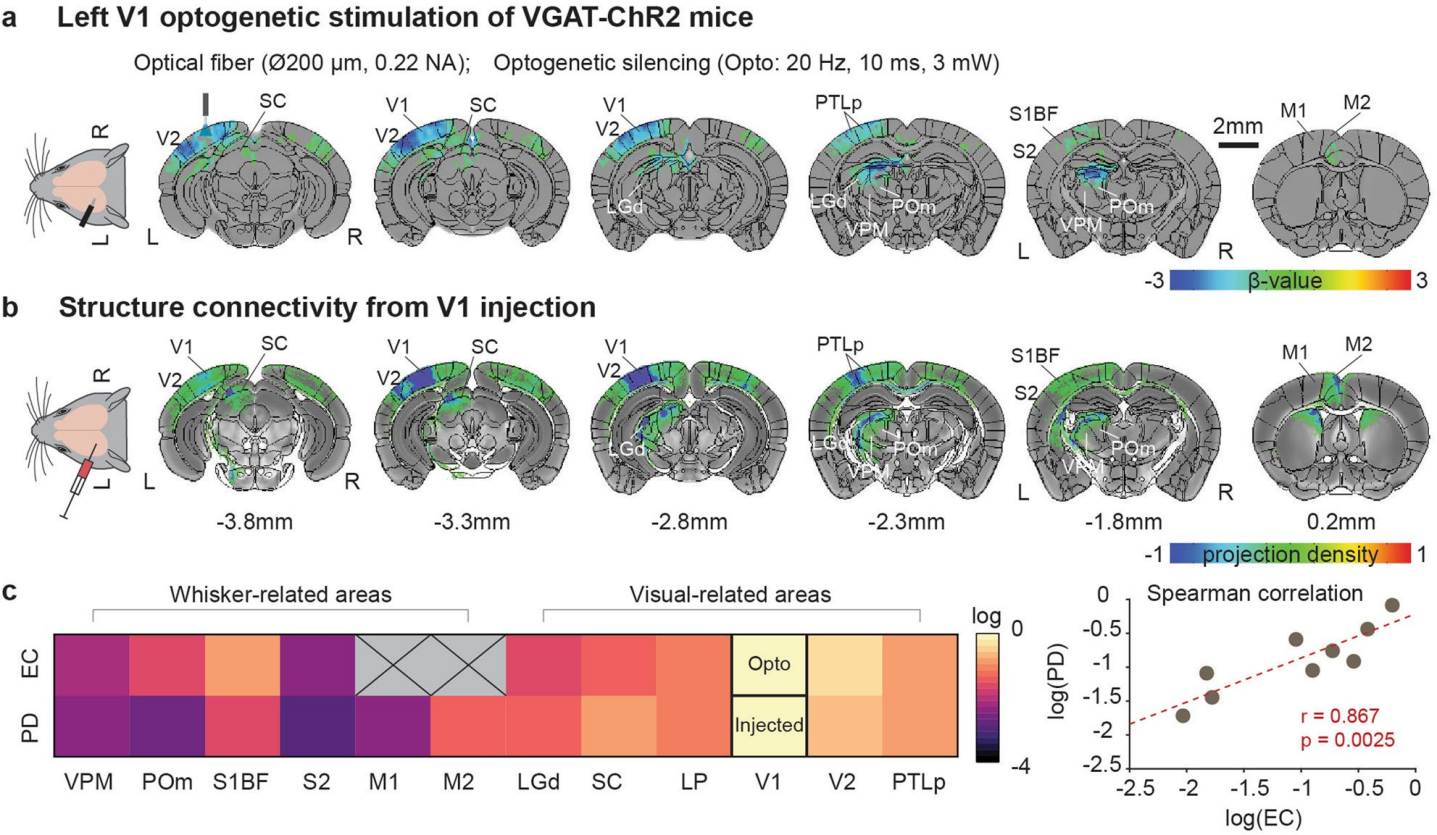
Comparison of functional effective connectivity by optogenetic fMRI (EC) and structure connectivity. (**a**) Brain-wide group BOLD fMRI maps to left V1 silencing overlaid on Allen Mouse Brain Atlas (n = 10; p < 0.005, FDR corrected). (**b**) Axonal projection maps from Allen Mouse Connectivity Atlas (https://connectivity.brain-map.org; experiment #307,321,674 for V1 injection). Areas are labeled, and the coronal slice positions are marked relative to Bregma. (**c**) Log-scale EC and projection density (PD). EC was not obtained from motor areas due to susceptibility artifacts. Spearman’s rank correlation analysis was performed between EC strengths and PD from V1 to all ROIs.

Visual-to-somatosensory enhancement in somatosensory whisker areas could arise from V1 and V1 downstream pathways involving higher-order visual cortex^6,8^. To investigate these potential V1-downstream circuits to somatosensory regions, we employed optogenetic silencing of excitatory neurons in V1. Activating ChR2-expressing inhibitory neurons in V1 suppresses cortical excitatory recurrent circuits and its outputs to downstream pathways^18,19,35,36^. Inactivating V1 with 20-s optogenetic stimulation of inhibition neurons resulted in negative BOLD changes at the ipsilateral V1, networked cortical visual regions (V2, PTLp) and subcortical visual sites (LGd, SC, LP) (Fig. 4a). Notably, a significant reduction was also observed in somatosensory-related thalamus VPM and POm, while only a slight change occurred in somatosensory cortices. It should be noted that due to image artifacts caused by susceptibility effects by residual blood clots or trapped air bubbles at motor areas, images in these regions exhibited significant distortions in some animals (see Supplementary Fig. 3) and were not included in further data analysis.

Optogenetic fMRI (ofMRI) of V1 silencing shows resting-state functional effective connectivity (EC) originated from V1, which may reflect structural connections. Thus, structural connectivity (SC) was constructed from Allen mouse data obtained with anterograde viral tracers that were focally injected into V1 (https://connectivity.brain-map.org/) (Fig. 4b). In general, ofMRI maps were topologically aligned with axonal projection density maps. To enable a quantitative comparison, fMRI responses and tracer counting were normalized by those in V1^37^ and presented as heatmaps in a log scale (Fig. 4c). The functional EC and axonal projection density were positively correlated with each other (Spearman coefficient ρ = 0.867, p = 0.0025). Notably, V1 is functionally and structurally connected to S1BF and weakly to VPM. However, the functional connection between V1 and POm exists without the monosynaptic anatomical connection, possibly due to the presence of polysynaptic connections^37–40^ involving other areas connected with V1 (see Supplementary Fig. 4).

### Dissection of visual-whisker pathways by sensory fMRI with V1 silencing

**Figure 5:**
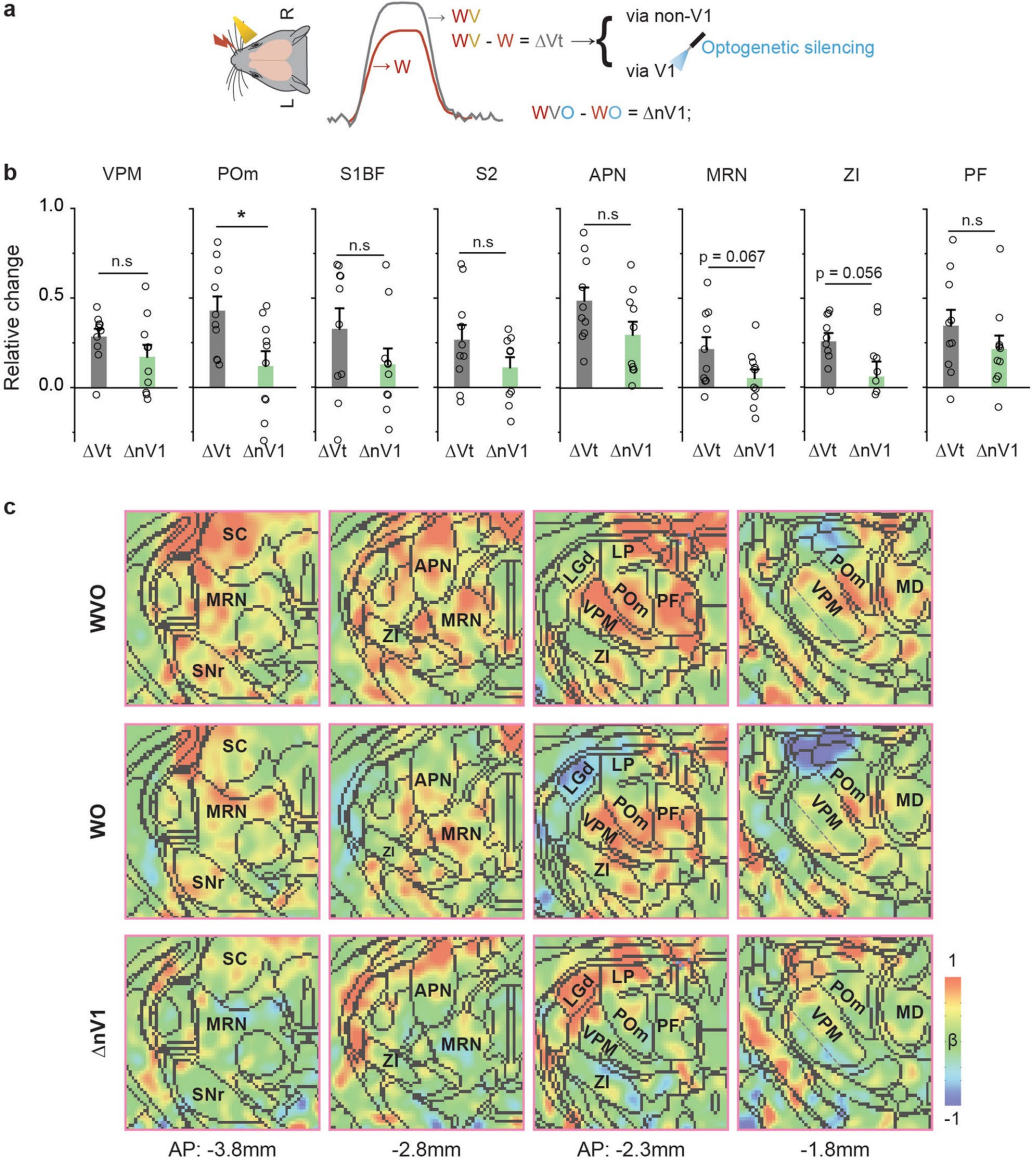
Optogenetic fMRI data for dissecting cross-modal pathways from visual inputs to whisker-related areas. (**a**) Schematics illustrating how to dissect potential pathways. The total enhancement in the whisker-related areas by visual stimulation was calculated as ΔVt = WV – W. Simultaneously silencing V1 was performed during bimodal stimulation (WVO) and whisker-only stimulation (WO). The non-V1 contribution, ΔnV1 = WVO – WO, was compared to ΔVt. (**b**) Functional contributions of non-V1 and V1 pathways to whisker-related regions, APN, MRN, ZI, and PF (n = 10 mice for each group). Error bars, SEM; n.s., not significant; and *p < 0.05 (two-sample t-test). (**c**) Non-thresholded group fMRI maps of WVO, WO and ΔnV1 overlaid on Allen Mouse Brain Atlas (n = 10 mice). Maps were zoomed in for better visualization, and coronal slice positions are marked relative to Bregma.

The total enhancement of whisker-related areal responses by bimodal stimulation (ΔVt = WV – W) can be attributed by both V1 and non-V1 pathways. To dissect these two contributions, we compared fMRI responses to whisker-visual bimodal stimulation with V1 silencing (WVO) and whisker-only stimulation with V1 silencing (WO). Since V1 silencing by itself induces negative fMRI signal changes in the stimulation site and in downstream regions (Fig. 4), the common suppression of resting-state connectivity should be removed by the difference between WVO and WO^19^, leaving only the non-V1 contribution of visual inputs, ΔnV1 = WVO – WO (Fig. 5a,c).

All fMRI responses in individual subjects were normalized to the BOLD response of whisker-only stimulation in S1BF to minimize inter-subject variations (Fig. 5b). The BOLD response from the visual inputs in VPM was predominantly originated from the non-V1 contribution (ΔnV1), as silencing of V1 did not significantly reduce the visual enhancement in the VPM. In contrast, the total visual enhancement in POm was reduced by > 75% due to V1 silencing (mean relative ΔVt = 0.43 versus ΔnV1 = 0.12); hence, the POm was mostly modulated by the V1 contribution. The whisker cortices (S1BF and S2) received inputs from both ΔVt and ΔnV1 (ΔVt = 0.33 versus ΔnV1 = 0.13 in S1BF; ΔVt = 0.27 versus ΔnV1 = 0.11 in S2) (Fig. 5b,c). Similarly, the APN and PF were partially reduced by V1 silencing, while the MRN and ZI were strongly inhibited by V1 silencing, indicating V1-driven inputs (Fig. 5b,c).

The flow of information from visual inputs to VPM appears to occur only through sparse axonal projections from specific visual cortical areas, V1 and the anterolateral visual area (VISal) (refer to Supplementary Fig. 4). However, the silencing of V1 does not have a significant impact on the visual enhancement of VPM during bimodal stimulation (ΔnV1 ~ ΔVt), suggesting that visual-related subcortical areas are the primary network for enhancing visual activity in VPM through unidentified intermediary regions. The information flow from V1 to POm (Fig. 5) must be conveyed via polysynaptic connections, as direct anatomical connections between V1 and POm are minimal (Fig. 4). Based on anatomical connections, possible candidates are SC, LP, and subregions in V2 including VISal and anteromedial visual area (VISam) (see Supplementary Fig. 4).

**Figure 6:**
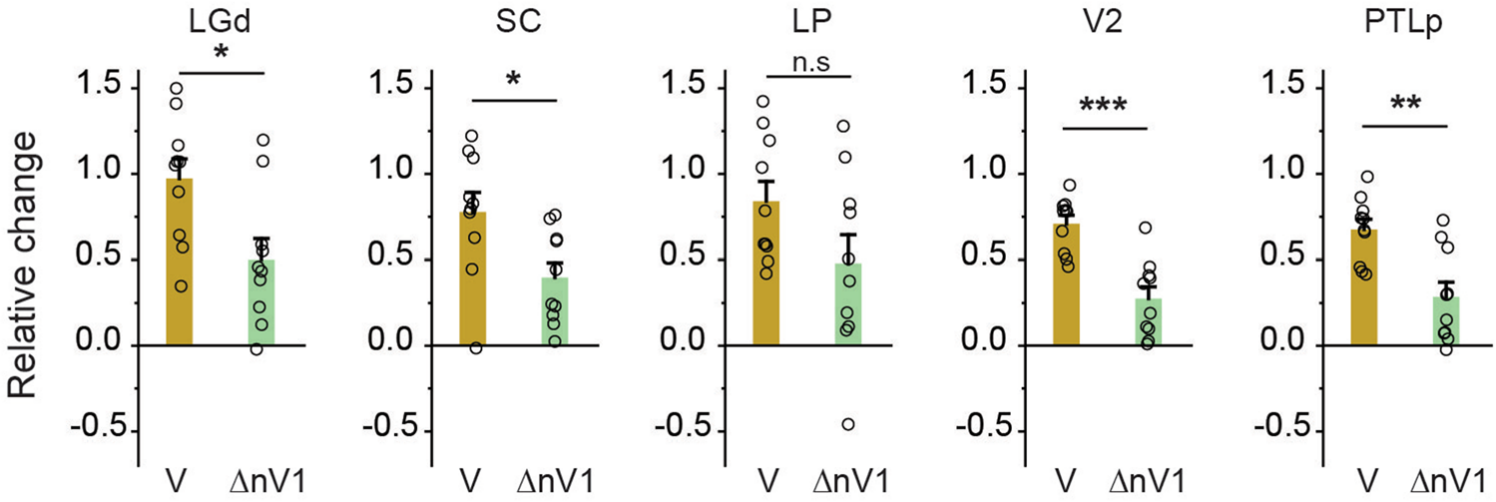
Total visual input and non-V1-related fMRI activity in visual areas during eye stimulation. Total visual fMRI activity was obtained from visual-only stimulation (V, n = 10 mice), and the non-V1 component (ΔnV1, n = 10 mice) was determined as the difference between fMRI data of simultaneously silencing V1 during bimodal stimulation (WVO) and whisker-only (WO). Error bars, SEM; *n.s.* not significant; *p < 0.05, **p < 0.01, ***p < 0.001 (two-sample t-test).

To further investigate possible intermediate visual areas related to POm connections (V1 → downstream subcortical areas/V2 → POm), the non-V1 contribution to visual areas was determined by the difference between WVO and WO (ΔnV1), while the V1 contribution was estimated by visual-only (V; see Fig. 1) minus ΔnV1 (Fig. 6). Firstly, LGd and PTLp received feedback and feedforward signals from V1 (V minus ΔnV1) and the non-V1 component (ΔnV1), respectively (Figs. 5c and 6 and for ΔnV1 map), but there are no direct anatomical connections from LGd and PTLp to POm/VPM (Supplementary Fig. 4). Thus, LGd and PTLp are unlikely to be involved in visual enhancement in POm/VPM. Secondly, SC also received both V1 and non-V1 contributions, while V2 was predominantly interacted with V1 (Fig. 6). Hence, these areas could serve as intermediate spots from V1 to POm through their axonal projection to POm (V1 → SC/V2 → POm) (see Supplementary Fig. 4).

## Discussion

In our high spatial resolution whole-brain mouse fMRI at 15.2 T, broad BOLD responses to different sensory stimulation were observed. Unimodal sensory stimulation not only activated the main pathway related to its specific function but also elicited responses that extended broadly to other sensory areas. The combination of whisker and visual stimulation revealed that whisker activity is strengthened by additional inputs from retina, while whisker stimulation only affects multisensory regions among visual-related areas. Employing V1 optogenetic inhibition can assist in delineating cross-modal pathways from visual inputs to whisker areas.

The presence of whisker-visual bimodal stimulation produced enhancements in BOLD responses of all whisker-related areas, suggesting that visual inputs assist the whisker network, a prominent sense in rodents used for navigating and exploring the environment^41–44^. Similar enhancements have been observed in several electrophysiology studies^1,3,9^. The enhancement of VPM activity might occur either directly through the VPM – V1 axonal connection or indirectly through the intralaminar thalamus^1,3^. In our data, VPM is dominantly affected by non-V1 signals, while POm is robustly modulated by V1 silencing. The exact pathway of non-V1 signals to VPM is unknown, which can be further investigated by circuit-specific silencing. Since fMRI activity in PF due to visual input was not significantly reduced by V1 silencing (Fig. 5), the visual input may be relayed to the parafascicular intralaminar nuclei through the SC^45^, then reaching VPM by a direct connection from PF to VPM^3^. On the other hand, the enhancement of POm activity by V1 might occur through SC^5^ and/or V2^46^. Since the ZI activity projects to POm during whisker activation^32^, the V1 effect may modulate POm response through SC/V2 → ZI → POm. To identify intermediate sites in the connection from V1 to POm, it is crucial to optogenetically silence V1-downstream circuits such as SC, V2, or ZI. The ZI also projects to MRN^47^, which is modulated by V1 inputs. The APN has been described as part of the visual pretectal complex as well as is involved in somatosensory modulation by recurrent projections from POm and SC^22,48,49^. These connections could lead to the non-V1 and V1 enhancement in APN during bimodal stimulation compared to unimodal stimulation.

The augmentation of the S1BF response might come from axonal cortico-cortical connection between S1BF and V1^7,9,50,51^ or from visual inputs to VPM and POm^5^. Subsequently, S2 could be affected by cortico-cortical connections with S1BF. Unlike rodents^9,30,51–55^, primates and humans exhibit a sparse direct connection between the somatosensory cortex and visual cortex^56–59^. Thus, the translatability of our findings may be limited. Nonetheless, our observations provide valuable insights for future studies focused on investigating the sources of visual integration in the whisker somatosensory network and exploring the roles of visual inputs in abnormal whisker-related behavior^60^.

There are several limitations in this study. Firstly, there was a distortion in the anterior brain image in the mouse motor areas (M1 and M2) due to susceptibility artifacts around the surgical area. This resulted in incomplete coverage of the somatosensory network in optogenetic fMRI. Secondly, multimodal integration may vary under different stimulus conditions. We used simple flashing light and electrical pad stimulations at a fixed strength. Exploring various stimulation strengths and/or different sites for stimulation (e.g., retinotopy, forepaw) could offer additional insights into multimodal integration. Additionally, a naturalistic paradigm, such as moving grating and air puff, is needed for further investigations. Thirdly, to validate our fMRI findings, it is necessary to conduct electrophysiological recordings studies. Electrophysiological recordings can offer additional insights that cannot be fully elucidated by BOLD fMRI, such as higher spatial and temporal multimodal integration. Fourthly, the integration of multiple senses becomes more complex under awake and behaving conditions. Since our studies were performed under anesthesia, further studies under awake conditions would be critical to understand multisensory integration.

In conclusion, we have successfully mapped visual and whisker networks and their interaction on a whole brain scale. Our findings indicate that both V1 and non-V1 pathways contribute to whisker somatosensory activity. The combination of sensory stimulation and focal silencing provides a viable fMRI approach for dissecting the functional contribution of specific brain areas or pathways.

## Methods

### Animal

In total, twenty male mice (23–32 g; 8–13 weeks old) were used in two different studies under ketamine-xylazine anesthesia^61^: (1) ten wildtype mice for visual and whisker stimulation BOLD fMRI (C57BL/6; Orient Bio, Seongnam, Korea), and (2) ten transgenic mice with ChR2 in inhibitory neurons (VGAT-ChR2-EYFP) for fMRI with cortical inactivation. The VGAT-ChR2-EYFP mice (B6.Cg-Tg(Slc32a1-COP4*H134R/EYFP)8Gfng/J) were bred in-house from breeding pairs obtained from Jackson Laboratory (Bar Harbor, ME, USA). Animals were housed in cages under controlled temperature and humidity conditions and a 12-h dark–light cycle with food and water provided ad libitum. All experiments were performed with approval by the Institutional Animal Care and Use Committee (IACUC) of Sungkyunkwan University following standards for humane animal care from the Animal Welfare Act and the National Institutes of Health Guide for the Care and Use of Laboratory Animals and ARRIVE guidelines 2.0 (Animal Research: Reporting in Vivo Experiments)^62^.

The fiber-optic cannulas (Thorlabs, Newton, New Jersey, USA) were implanted^63^ in transgenic mice > 2 weeks before fMRI experiments. The optical fiber cannula (200 µm inner core diameter, NA = 0.22) was implanted into the left V1 (AP:—3.64 mm, ML: + 2.3 mm relative to bregma and DV: + 0.2 mm relative to the surface).

For stereotactic surgical and BOLD fMRI, mice were initially anesthetized with 4% isoflurane in 20% O_2_/80% air mixture then given an induction dose with a mixture of ketamine (Yuhan, Korea) and xylazine (Rompun^®^, Bayer, Korea) (100/10 mg/kg for initial IP, and 25/1.25 mg/kg intermittent IP injections every 30–45 min) under self-breathing through a nose cone that provided a continuous supply of oxygen and air (1:4 ratio) at a rate of 1 L/min (SAR-1000, CWE, Ardmore, USA or TOPO, Kent Scientific Corporation, Torrington, CT, USA) to maintain an oxygen saturation level > 90%^20,61,63,64^. To minimize whisker movements during fMRI studies, all whiskers were neatly trimmed before the animal was inserted into the magnet. Temperature was monitored with a rectal probe and maintained at ± 37 °C via warm-water circulation pad. Respiration and heart rate were monitored with a pressure detector placed on the ventral surface and pulse oximeter (SA Instruments, Inc., Stony Brook, NY, USA) placed on the tail, respectively. Physiological information was monitored using a data acquisition system (Acknowledge, Biopac Systems, Inc., Goleta, CA, USA).

### Sensory stimulation

For visual stimulation to right eye, optic fibers with 0.5-mm diameter were placed 2 cm distally from right eye of the animal and connected to a cold white LED driver (DC200, Thorlabs, Newton, New Jersey, USA). The illuminance of the LED light was measured to be around 10 lx at the fiber tip. The LED driver was controlled by a pulse generator (Master 9; World Precision Instruments, Sarasota, FL, USA). Stimulation parameters for visual stimuli were at a frequency of 5 Hz, pulse width of 10 ms^15^. For whisker stimulation, we opted for electric stimulation instead of a mechanical stimulator due to its ease of implementation within a small-bore size magnet. Whisker pad electric stimulation has consistently induced fMRI and neural responses in whisker-related somatosensory areas (e.g., VPM, S1, S2)^22–24,65^, making it a suitable choice for whisker stimulation. For right whisker-pad electrical stimulation, a three-pin electrode pad was placed on the mouse’s right whisker-pad. Stimulation parameters for whisker stimuli were at a frequency of 4 Hz, pulse width of 0.5 ms, and current intensity of 0.6 mA^22^. Ten C57BL/6 mice were used for right eye stimulus only (V), right whisker stimulation only (W), and bimodal stimulation with combination of right whisker and right eye stimulus simultaneously (WV).

### Optogenetic silencing of V1

The experimental procedure for optogenetic fMRI (ofMRI) was previously described in detail^63,66^. Light was delivered to the left V1 through a fiber-optic cable (length: 7 m, Doric Lenses Inc, Quebec, Canada) coupled to a 473-nm diode-pumped solid-state laser (Changchun New Industries Optoelectronics Tech. Co., Ltd, Changchun, China). The constant output power was calibrated to be 3 mW at the tip of the optic fiber, as measured by a power meter (PM100D, Thorlabs, USA). The optical stimulation was then given at 20 Hz/10 ms parameter to silence excitatory pyramidal neurons through optogenetic activation of interneurons of VGAT-ChR2 mice^19,40^. To block undesired activation of the visual pathway by light leakage, the connection between the fiber optic cable and the implanted cannula was covered carefully. Ten VGAT-ChR2 mice were used to perform optogenetic fMRI for cortical silencing^18,19,40^. Four different stimulation data were obtained: V1 optogenetic silencing (O), right whisker stimuli with and without left V1 inhibition (W, WO), and right whisker + right eye + left V1 inhibition (WVO).

### fMRI experiments at 15.2 T

All MRI experiments were performed on a 15.2-T MRI scanner with an actively shielded 6-cm diameter gradient operating with a maximum strength of 100 G/cm and a rise time of 110 µs (Bruker BioSpec MRI, Billerica, MA, USA). A 15-mm ID customized surface coil for radiofrequency (RF) transmission/reception was centered on the imaging slices covering the whole brain, and the animal was positioned to make the brain close to the isocenter of the magnet.

Magnetic field homogeneity was shimmed globally using the field map method. Eighteen contiguous 0.5-mm thick coronal slices T1-weighted anatomical images were acquired using a fast-low angle shot (FLASH) sequence with field of view (FOV) = 15.6 × 7.8 mm^2^, matrix size = 256 × 128, in-plane resolution = 0.061 × 0.061 mm^2^, repetition time (TR) = 384 ms, echo time (TE) = 3.34 ms, and number of averages (NA) = 4. Before starting the fMRI experiments with an EPI sequence, local field homogeneity was optimized in the ellipsoidal volume covering the sensory areas using the Bruker MAPSHIM shimming protocol (ParaVision 6, Bruker BioSpec, Billerica, MA, USA). All fMRI data were acquired using single-shot gradient-echo EPI with eighteen contiguous slices without gaps in the coronal plane, FOV = 15.6 × 7.8 mm^2^, acquisition matrix = 106 × 53, in-plane resolution = 0.147 × 0.147 mm^2^, slice thickness = 0.5 mm, TR/TE = 1000/11 ms, flip angle = 50°, NA = 1, sampling bandwidth = 300 kHz, and 15 dummy scans. Each fMRI trial consisted of a 40-s pre-stimulus, 20-s stimulus, 60-s inter-stimulus, 20-s stimulus, and 60-s post-stimulus. 8–10 fMRI trials were obtained for each stimulus condition on each animal. Image acquisition and stimulus application were synchronized.

### Functional imaging data analyses

All data analyses were performed with the Analysis of Functional NeuroImages package^67^, the FMRIB Software Library^68^, Advanced Normalization Tools^69^, and Matlab^®^ codes (MathWorks, Natick, MA, USA). All quantitative values are represented as the mean ± standard error of the mean (SEM). The statistical significance of the results was assessed according to a p < 0.05 criterion.

Each functional image volume underwent preprocessing steps, including slice timing correction and motion correction by realignment to the first volume. Subsequently, the preprocessed data within the same session were averaged, and linear detrending was applied to mitigate signal drift. Time course normalization was performed by using the average of the baseline volumes before spatial normalization to a general brain template. The spatial normalization was conducted using the following procedure. Firstly, multislice functional EPI images were co-registered to the anatomical T1-weighted images from the same subject. Secondly, the T1-weighted images of all subjects were normalized and averaged by applying linear and nonlinear transformations to generate a mouse brain template. Thirdly, all EPI images co-registered in the first step were normalized to the mouse brain template (50 × 50 × 50 µm^3^) using the co-registration parameters obtained in the second step.

The Allen Mouse Brain Atlas was also registered to the brain template. In that common brain space, spatial smoothing was applied using a Gaussian kernel with a 147-µm full-width at half maximum (FWHM of 1 pixel). Animal-wise functional maps were made by a GLM analysis using a stimulation paradigm convolved with a double-gamma variate hemodynamic response function (HRF). The beta value was obtained from the GLM analysis, which represents the estimated effect size. After that the group activation maps were generated using a one-sample t-test and considering the significance of the false discovery rate (FDR) corrected to p < 0.01, then overlaid on the brain template. Statistical values were reported in the figure.

### Quantitative BOLD ROI analyses

Regions of interests (ROIs) were defined based on the Allen Mouse Brain Atlas (Allen Institute for Brain Science, http://mouse.brain-map.org/)^13^. All the ROIs considered as related to the visual pathways and somatosensory networks were selected based on the brain atlas: LGd dorsal lateral geniculate thalamic nucleus, SC superior colliculus, LP lateral posterior thalamic nucleus, V1 primary visual cortex, V2 higher-order visual cortex, PTLp posterior parietal association areas, VPM ventral posteromedial thalamic nucleus, POm posterior medial thalamic nucleus, S1BF primary somatosensory barrel field, S2 secondary somatosensory area, M1 primary motor area, M2 secondary motor area, anterior pretectal nucleus (APN), midbrain reticular nucleus (MRN), substantia nigra (SNr), zona incerta (ZI), parafascicular nucleus (PF), and mediodorsal thalamic nucleus (MD).

Two ROI analyses were performed. (1) The averaged beta value within each ROI was calculated from individual animal beta maps obtained from the GLM analysis of unimodal sensory stimulation. (2) BOLD time courses of ROIs were extracted from the fMRI data without spatial smoothing applied for each mouse. The BOLD fMRI responses of the two stimulus blocks were averaged in each animal. Then mean percent signal changes were calculated excluding the initial 3–6 s of data after stimulus onset to avoid the initial transition period.

### Structural connectivity analyses

For the structural connectivity data, experiment #100141598, 112827164, 146078721, 266585624, 307321674 (for V1 injection), 146077302, 116903968, 120437703, 100,148,503, and 286301303 were obtained from the Allen Mouse Connectivity Atlas (https://connectivity.brain-map.org) and presented as projection density (PD). PD was calculated as the projection density in each ROI divided by the density of the source (e.g., V1) (Fig. 4 and Supplementary Fig. 4)^37^. To compare the structure connectivity with the effectivity connectivity (EC) of V1 silencing, beta values from the GLM analysis were averaged among all voxels within each ROI in each animal. EC was computed as a sum of voxel-by-voxel responses (beta values) in each ROI divided by a sum of voxel-by-voxel responses of the V1 (Fig. 4)^37^. Both PD and EC were presented on a log scale. Spearman's rank correlation analysis was performed between PD and EC^37^. The presence of EC was determined by a statistical test (one-sample t-test against zero; p < 0.05), and effective connectivity was not obtained from motor areas due to susceptibility artifacts. Projection volumes < 0.01 µm^3^ (voxel volume) were removed.

### Supplementary Information

Supplementary Figures.

## Data Availability

The unprocessed functional and anatomical data are available from the corresponding author upon request. They will be provided in NIFTI format. The preprocessing scripts can also be shared upon request.
