# Supplementary material for: Separation of bimodal fMRI responses in mouse somatosensory areas into V1 and non-V1 contributions
Source: Sci Rep. 2024 Mar 15;14:6302. doi: 10.1038/s41598-024-56305-w (PMC10943206; doi:10.1038/s41598-024-56305-w)
Supplement: Supplementary file 1 — Supplementary Figures. [file 41598_2024_56305_MOESM1_ESM.pdf]

Separation of bimodal fMRI responses in mouse somatosensory areas into V1 and non-V1 contributions

Thi Ngoc Anh Dinh[1, 2], Hyun Seok Moon[1, 2], Seong-Gi Kim[1, 2, 3, *]

[1] Center for Neuroscience Imaging Research (CNIR), Institute for Basic Science (IBS), Suwon, 16419, South Korea.

[2] Department of Biomedical Engineering, Sungkyunkwan University, Suwon, 16419, South Korea.

[3] Department of Intelligent Precision Healthcare Convergence, Sungkyunkwan University, Suwon, 16419, South Korea.

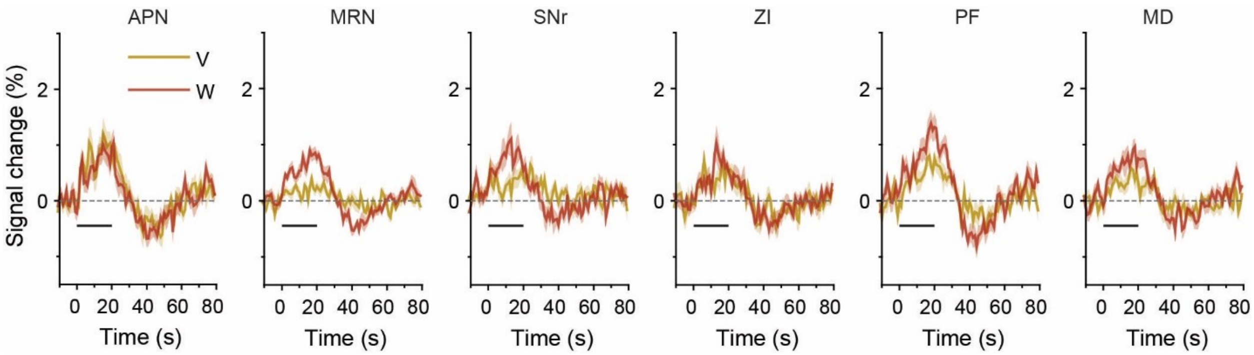

Supplementary Figure 1. Averaged time courses of subcortical ROIs responding to unimodal visual (V) and whisker (W) stimulation. Error bars: SEM (n =10 mice); black lines indicate 20-s stimulation period.

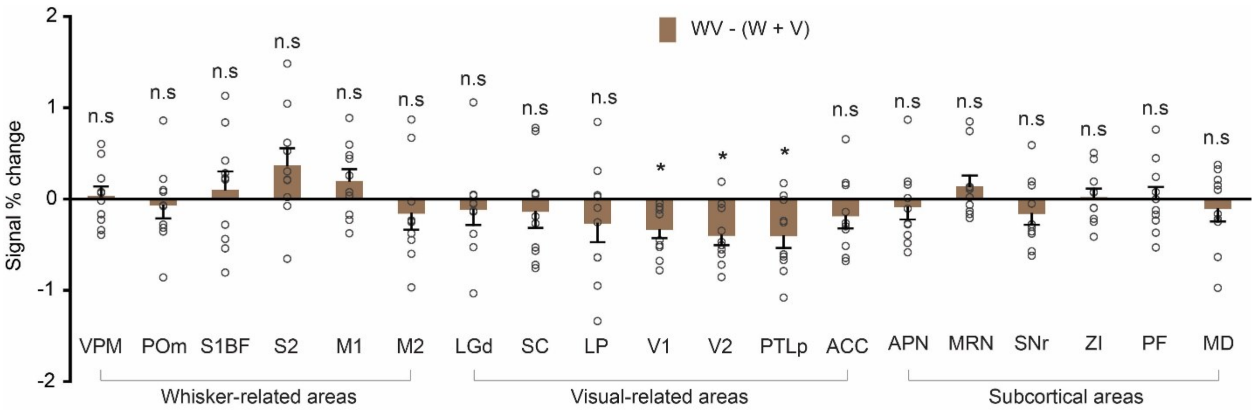

Supplementary Figure 2. Difference between bimodal responses [WV] and the sum of unimodal responses ([W]+[V]). A value close to 0 indicates that bimodal responses can be well explained by an additive model. V1, V2 and PTLp have significant negative values corresponding to a sub-additive model indicative of response depression. Error bars, SEM; n.s., not significant; *$p < 0.05$ (one sample t-test against 0).

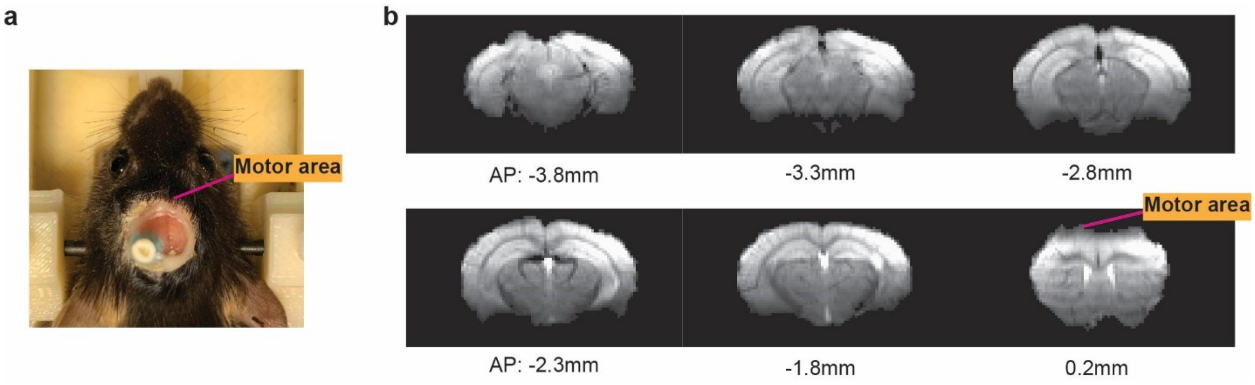

Supplementary Figure 3. Fiber implementation and its effect on image quality. (a) A picture of a mouse with an optogenetic fiber in the animal cradle (ivory frame with two black ear bars). The muscle above the skull was removed, exposing the dorsal brain. Optic fiber was secured with dental cement. (b) Raw EPI images of one representative animal shows artifacts caused by the susceptibility effect in the motor areas.

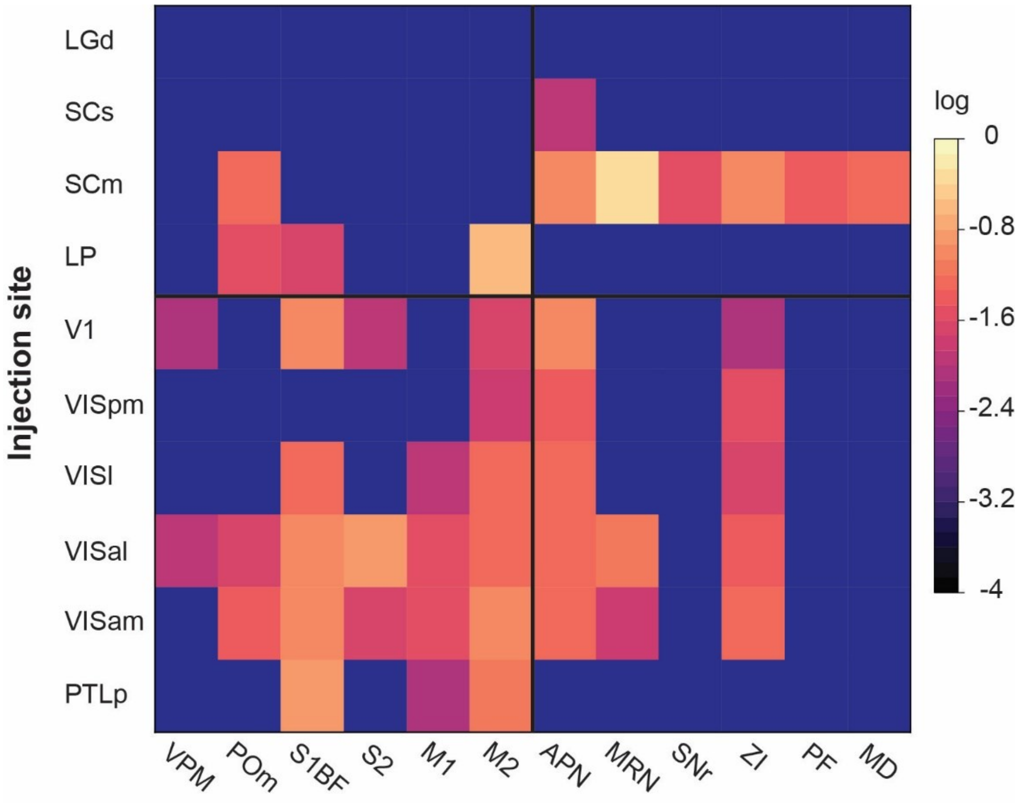

Supplementary Figure 4. Log-scale of axonal connectivity projection density from visual ROIs to whisker regions imported from Allen Mouse Brain Connectivity Atlas (https://connectivity.brain-map.org/; Experiment #: 100141598, 112827164, 146078721, 266585624, 307321674, 146077302, 116903968, 120437703, 100148503, 286301303).
